# Analysis of the Regionality of the Number of Tweets Related to the 2011 Fukushima Nuclear Power Station Disaster: Content Analysis

**DOI:** 10.2196/publichealth.7496

**Published:** 2018-12-18

**Authors:** Tomohiro Aoki, Teppei Suzuki, Ayako Yagahara, Shin Hasegawa, Shintaro Tsuji, Katsuhiko Ogasawara

**Affiliations:** 1 Graduate School of Health Sciences Hokkaido University Sapporo Japan; 2 Faculty of Health Sciences Hokkaido University Sapporo Japan; 3 Department of Radiological Technology Hokkaido University of Science Sapporo Japan

**Keywords:** Fukushima nuclear disaster, Twitter messaging, radiation, radioactivity, radioactive hazard release, geographic location, information dissemination

## Abstract

**Background:**

The Great East Japan Earthquake on March 11, 2011, triggered a huge tsunami, causing the Fukushima Daiichi nuclear disaster. Radioactive substances were carried in all directions, along with the risks of radioactive contamination. Mass media companies, such as television stations and news websites, extensively reported on radiological information related to the disaster. Upon digesting the available radiological information, many citizens turned to social media, such as Twitter and Facebook, to express their opinions and feelings. Thus, the Fukushima Daiichi nuclear disaster also changed the social media landscape in Japan. However, few studies have explored how the people in Japan who received information on radiation propagated the information.

**Objective:**

This study aimed to reveal how the number of tweets by citizens containing radiological information changed regionally on Twitter.

**Methods:**

The research used about 19 million tweets that included the terms “radiation,” “radioactivity,” and “radioactive substance” posted for 1 year after the Fukushima Daiichi nuclear disaster. Nearly 45,000 tweets were extracted based on their inclusion of geographic information (latitude and longitude). The number of monthly tweets in 4 districts (Fukushima Prefecture, prefectures around Fukushima Prefecture, within the Tokyo Electric Power Company area, and others) were analyzed.

**Results:**

The number of tweets containing the keywords per 100,000 people at the time of the casualty outbreak was 7.05 per month in Fukushima Prefecture, 2.07 per month in prefectures around Fukushima Prefecture, 5.23 per month in the area within Tokyo Electric Power Company, and 1.35 per month in others. The number of tweets per 100,000 people more than doubled in Fukushima Prefecture 2 months after the Fukushima Daiichi nuclear disaster, whereas the number decreased to around 0.7~0.8 tweets in other districts.

**Conclusions:**

The number of tweets per 100,000 people became half of that on March 2011 3 or 4 months after the Fukushima Daiichi Nuclear Plant disaster in 3 districts except district 1 (Fukushima Prefecture); the number became a half in Fukushima Prefecture half a year later.

## Introduction

### Fukushima Daiichi Nuclear Disaster

On March 11, 2011, the Great East Japan Earthquake struck off the coast of Tohoku, bringing a huge tsunami that brought catastrophic destruction along the Pacific-facing coast of Tohoku and Kanto regions, causing the Fukushima Daiichi nuclear disaster. As a result, a large quantity of radioactive materials leaked, causing radioactive pollution of the water. The radiation levels caused by the Fukushima Daiichi nuclear disaster threatened not only human health but also agriculture and fishing industry. Further, it had psychological impacts on the long-term refugees forced to leave their homes within the “difficult-to-return zone” or “restricted residence zone” in the areas surrounding the Fukushima Nuclear Power Plant.

### Information Diffusion

Soon after the disaster, public opinions are formed through various platforms including social network services (SNS) [1]. The Fukushima Daiichi nuclear disaster was reported immediately by the mass media, including newspapers, TV stations, and internet news sites. Citizens witnessed the terrible sight of the nuclear plant disaster and learned the radiation dose in various areas, along with other information on radiation. Many expressed their emotions and opinions related to the nuclear plant disaster and radiation as well as shared information on the same using SNS, such as Twitter and Facebook. Therefore, information spread rapidly.

The information sharing on social media had far-reaching positive impacts, including real-time property and high diffusibility. Thus, consumers of information are simultaneously contributors of information [2]. However, it became a problem at the Fukushima Daiichi nuclear disaster that the information that spread rapidly included misleading reports such as claims that iodine is useful for treating radioactivity as a replacement of stable iodine. Stable iodine is used for thyroid exposure reduction under a doctor’s prescription, but iodine was used for a person who did not have to take it. Taking in a toxic substance included in iodine and iodine in large quantities caused a health risk. People need to obtain correct information quickly in times of disasters, such as the Fukushima Daiichi nuclear disaster. As mentioned above, however, incorrect information on the radiation spread rapidly as well.

For confirmed truths and false rumor propagation in social media, false rumors tend to receive more questions; thus, it has been reported that it is possible to distinguish between them [3]. In addition, it has been reported that inaccurate information on social media is later modified by other users, so harmful and incorrect rumors are not particularly enhanced by using social media [4].

However, to distinguish between confirmed truths and false rumors, it is necessary to gather a lot of data using aggregate analysis of social media. Real-time false information and rumors at the time of a disaster require time to be modified by confirmed truths, so it is expected that they will possibly lead to temporary confusion and harm. The spread of wrong information on radiation was regarded as a problem in the Fukushima Daiichi nuclear disaster. Incorrect information needs to be addressed to ensure that citizens are not confused when a disaster such as the Fukushima Daiichi nuclear disaster occurs. We believe that it is necessary for citizens to get accurate medical information quickly in the event of a catastrophe.

### Twitter

Twitter was the largest microblogging service, with about 200 million users, as of March 2011 [5]. Twitter is an information service through which users can post short messages called “tweets.” Tweets are short but condensed personal messages with a 140-character limit designed for rapid reporting from mobile devices [6]. In Twitter, for the purpose of reading tweets, it is necessary to follow the users. Thus, the users can read tweets posted by followees (following users) and propagate information by showing the timeline of the follower (followed users) tweets posted by oneself. Posted tweets are displayed on the follower’s timeline in a chronological order and are updated dynamically. Thus, tweets can be read by several followers immediately as they are posted.

**Figure 1:**
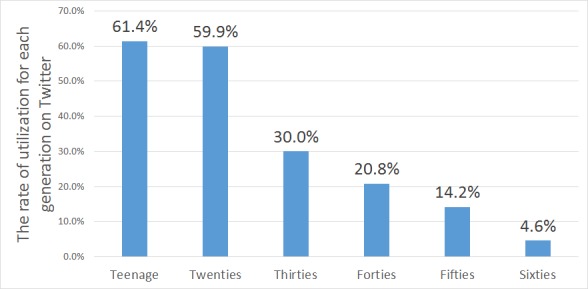
Rate of utilization for each generation on Twitter in Japan.

Information on Twitter is characterized by its real-time availability and high information propagation power. As tweets must be not more than 140 characters in length, posting is easier compared with other SNS types. Users can post daily events and random thoughts as well as obtain regional information immediately. Compared with other SNS types, approval is unnecessary for following relations on Twitter, and information can be acquired easily according to one’s interest except where a user opts to maintain a private timeline. In addition, information spreads easily through the “retweet” function that enables users to quote others’ tweets. Other features include embedding of geographic information (latitude and longitude) in tweets and posting using “bots” programs that enable automatic and scheduled posting. Geographic information will be attached to a tweet only if that user permits sending location information. Figure 1 shows the rate of utilization for each generation on Twitter [7]. Teenagers are the largest group with available access to Twitter, where availability tends to decrease with age. The usage rate of Twitter varies according to age, and as the age increases, the utilization rate decreases. The limitations of Twitter research, in general, are to gather a lot of tweets with geographical information and to surely collect the tweets of all ages. Therefore, it is difficult for Twitter analysis to grasp the information dissemination situation of all ages.

**Figure 2:**
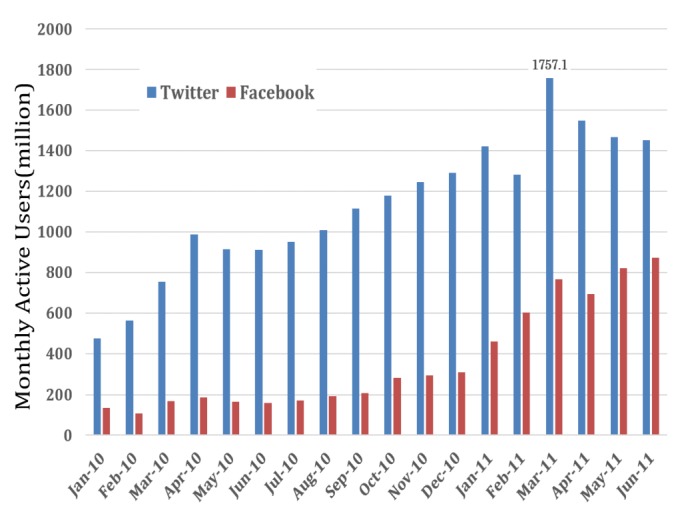
The number of active users on Twitter and Facebook.

Twitter, launched in July 2006, began to be used in Japan on April 23, 2008. The number of Japanese users increased rapidly after new mobile sites were established across Japan in October 2009. Figure 2 shows the changes in the number of Twitter users and Facebook users in Japan [5,8]. The active user is defined as a monthly active user on Twitter. In March 2011, the average daily number of tweets reached about 18 million, and Twitter was used for safety confirmation and information exchange in the aftermath of the Fukushima Daiichi nuclear disaster as conventional information and communication infrastructures suffered severe damage.

Mendoza et al analyzed Twitter information immediately after the occurrence of 2010 Chile earthquake. As a result, it became clear that the most tweets were generated immediately after the earthquake occurred [3]. Qu et al conducted information analysis on the disaster sent by the Chinese microblogging site Sina Weibo after the occurrence of Yushu earthquake in 2010. It became clear that disaster-related situation update messages were the second most generated tweets[9]. Acar et al reported that people indirectly influenced by the Great East Japan Earthquake in 2011 had been tweeting about indirect and future outcomes of the earthquake, including nuclear plant disaster-associated risks [10]. These three papers indicate that tweets on nuclear disaster were transmitted as situation update information by those who were affected by indirect earthquakes immediately after the earthquake occurred. Also, it is conceivable that the number of tweets will increase as one gets closer to the disaster occurrence area. Acar et al have reported that people affected by direct disasters tend to tweet survival-related topics [10]. People who were influenced by the direct earthquake would have thought that nuclear disaster information is a survival-related topic after information on nuclear plant disaster-associated risks was transmitted to disaster occurrence areas through Twitter and media. As a result, it seems that information on radioactive contamination increased even in areas affected directly. By this means, tweets on radioactive contamination are considered to cause regional differences. As for the information on radioactive contamination, it is expected that there will be a change in the amount of information over time as well as a regional difference in information, such as that in the tweet information that has occurred in the case of a big earthquake so far. However, after the occurrence of a disaster, analyses of changes in the number of tweets on regional radiation information and regional differences have not been conducted.

### Objective

Xin Lu and Christa Brelsford analyzed tweets from February 28 to March 7, 2011 (before the Tohoku earthquake on March 11, 2011) and from March 14 to March 21, 2011 (after the earthquake), reporting distinctive changes in patterns of interactions in Web-based communities that had been affected by a natural disaster compared with communities that had not been affected [11]. Thomson et al analyzed tweets with the hashtag #fukushima, reporting that close to 70% of synthesis-derivative tweets (whereby tweets and other third-party-sourced information is passed on wholesale) were based on highly credible sources [12,13]. However, at the time of the disaster, there were few studies that investigated the temporal change in tweet number and its regionality with respect to radiation emitted among Japanese people. This study aimed to reveal how the number of tweets by citizens containing radiological information changed regionally on Twitter.

## Methods

### Research Objects

We analyzed 45,829 tweets, extracted from approximately 19 million tweets that contained any or all of the terms “radiation,” “radioactivity,” and “radioactive materials” and that were posted from 0:00 on March 11, 2011, to 23:59 March 10, 2012, on Twitter, as research objects. These tweets were chosen based on containing latitude and longitude information. The total population in each prefecture was used for the total population in each district [14].

### Classification of Districts

**Figure 3:**
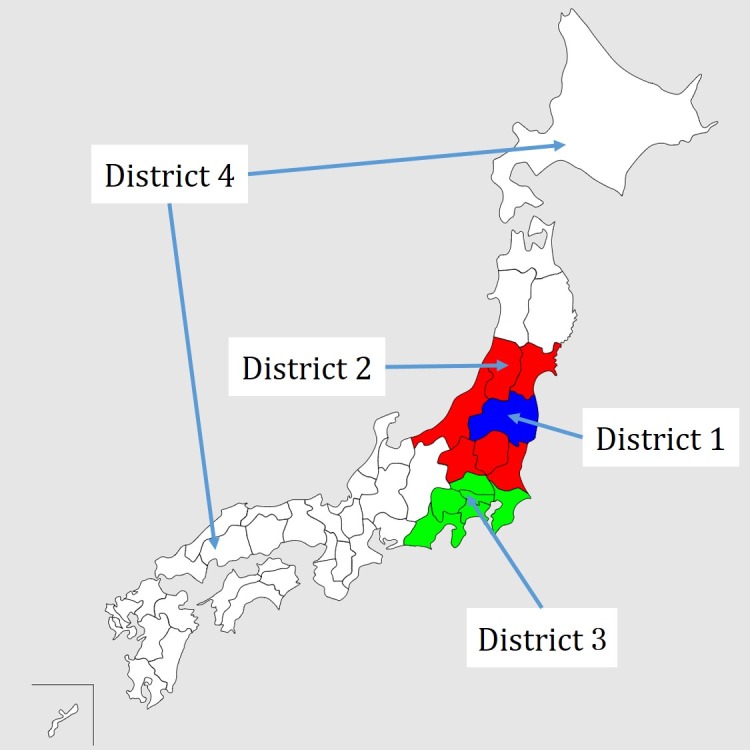
Locations of the districts of Japan classified according to trend analysis of Twitter after the Great East Japan Earthquake.

Japan was classified into 5 districts in reference to use trend analysis of Twitter after the Great East Japan Earthquake [15]. Table 1 and Figure 3 show the definition and classification of the districts.

Fukushima Prefecture with the Fukushima Nuclear Power Plant was the catastrophic area set as district 1. The prefectures around district 1 were the damaged areas categorized under district 2. The prefectures receiving electric power supply from Tokyo Electric Power Company, excluding district 1 and 2, were the indirectly damaged areas included in district 3. The prefectures outside districts 1 to 3 were the nondisaster areas set as district 4. Finally, areas outside Japan were the other areas categorized as district 5. The study specified the places where tweets were posted according to their geographic information (latitude and longitude). Usoinfo reverse geocoder version 1.1 software was used to convert latitude and longitude information into the address of a corresponding point [16]. The districts where tweets were posted were then specified according to their addresses, and their distribution was mapped. This study did not use the tweets in district 5.

### Comparison of Number of Tweets per 100,000 People in Each District

This study compared the number of tweets per 100,000 people every 1 month to solve the problem that the population was different in each district. In each district, the number of tweets in 1 month in a population of 100,000 people was counted using the total population and number of tweets in 1 month in each district. A month was defined as a 30-day period, beginning from 00:00 of March 11 to 23:59 of the 29th day; that is, the second month started at 0:00 of April 11 and so on. The study comprised 3 steps. First, we visualized how the number of tweets per 100,000 people in each district changed with each passing month after the Fukushima Nuclear Plant disaster. The districts were then compared in terms of tweeting trends. Second, we excluded bots to compare only the tweets posted by actual citizens for each district and visualize the changes in the civic interest toward radiation. Third, we compared the number of tweets based on that of tweets in March in each district shortly after the start of the Fukushima Nuclear Plant disaster. The relative number of tweets every month was then calculated to express the increase and decrease in the number of tweets. The percentages informed a visualization of how the number of tweets in each district changed after the Fukushima Nuclear Plant disaster, including the tweet to population ratio.

**Table 1:** Definition and classification of the districts of Japan according to trend analysis of Twitter after the Great East Japan Earthquake.

District	Definition	Prefectures
District 1	Catastrophic area (Fukushima Nuclear Power Plant location)	Fukushima
District 2	Damaged area (Prefectures around Fukushima)	Miyagi, Yamagata, Ibaraki, Gumna, Niigata, Tochigi
District 3	Indirectly damaged area (Prefectures in Tokyo Electric Power Company except district 1 and 2)	Saitama, Chiba, Tokyo, Kanagawa, Yamanashi, Shizuoka
District 4	Nondisaster area (Prefectures except district 1-3)	Other prefectures
District 5	Foreign countries and the sea	N/A^a^

^a^N/A: not applicable.

## Results

### Total Population and the Number of Tweets in Each District

Table 2 shows the number of tweets and total population in each district. Number of tweets was most numerous in district 3 (indirectly damaged area), and population size was greatest in district 4 (nondisaster area). Furthermore, tweets per population was highest in district 1 (catastrophic area).

### Comparison of Number of Tweets per 100,000 People in Each District

**Figure 4:**
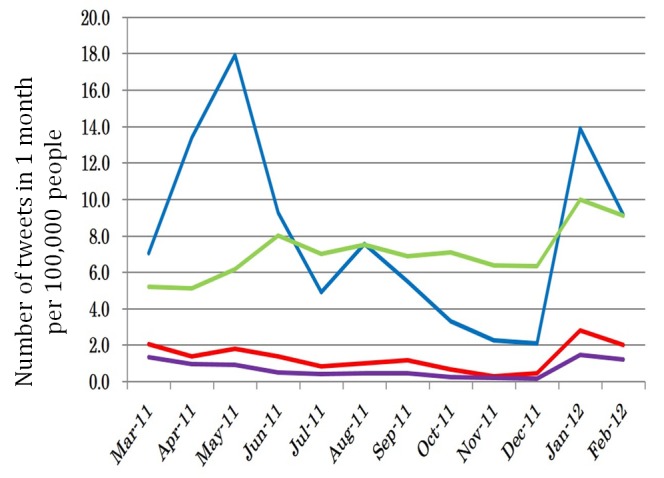
Number of tweets per 100,000 people in each district according to trend analysis of Twitter after the Great East Japan Earthquake.

Figure 4 shows the number of tweets per 100,000 people in each district. In district 1 (blue line), the number of tweets rose rapidly shortly after the Fukushima Nuclear Plant disaster outbreak. In district 3 (green line), the trend was a gentle rise throughout the year after the Fukushima Nuclear Plant disaster outbreak. In district 2 (red line) and 4 (purple line), the number of tweets decreased gradually throughout the year after the Fukushima Nuclear Plant disaster outbreak. Meanwhile, all districts showed an increase in the number of tweets in January 2012.

**Table 2:** Number of tweets and total population in each district according to trend analysis of Twitter after the Great East Japan Earthquake.

District name	Number of tweets	Population	Tweets per population (%)
District 1	1956	2,029,064	0.10
District 2	2042	12,877,060	0.02
District 3	34,152	40,246,646	0.08
District 4	6136	72,904,582	0.01
District 5	1543	N/A^a^	N/A

^a^N/A: not applicable.

**Figure 5:**
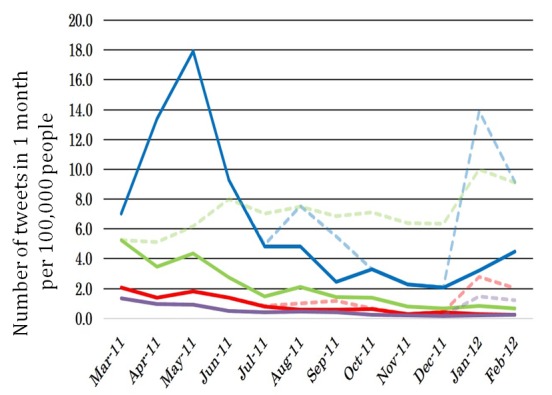
Number of tweets per 100,000 people in each district according to trend analysis of Twitter after the Great East Japan Earthquake.

Figure 5 shows the number of tweets per 100,000 people in each district. Tweets posted automatically were removed. The dotted lines of the graph express the number of all tweets per 100,000 people, whereas the solid lines express the number of tweets excluding those posted automatically. A solid line and a dotted line separated by a distance indicate that the percentage of tweets posted automatically had the majority in a certain district. In district 1 (blue lines), the dotted and solid lines overlap each other at the point where the number of tweets increased rapidly after the occurrence of first Fukushima Nuclear Power Plant disaster. Therefore, the number of tweets increased in this period because the number of tweets posted by citizens increased. Meanwhile, in district 3 (green lines), the number of all tweets per 100,000 people increased through the year, but the number of tweets excluding those posted automatically decreased after the Fukushima Nuclear Plant disaster. As such, the number of tweets increased in district 3 because of automatically posted tweets, but the ratio of tweets posted by citizens decreased gradually.

### Comparison of the Relative Number of Tweets in Each District

**Figure 6:**
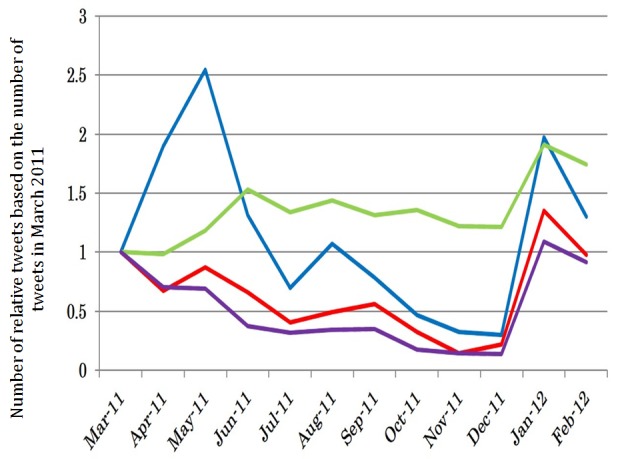
Ratio of the number of tweets in each month based on the number of tweets in each district at the time of Fukushima Nuclear Plant disaster.

Figure 6 shows the ratio of the number of tweets in each month based on the number of tweets in each district when the Fukushima Nuclear Plant disaster occurred. In district 1 (blue line), the number of tweets increased to about 2.5 times 2 months after the Fukushima Nuclear Plant disaster outbreak. Meanwhile, in districts 2 (red line) and 4 (purple line), the number of tweets decreased slowly after the Fukushima Nuclear Plant disaster outbreak. District 3 (green line) showed an increase throughout the year. All districts showed an increase in the percentage of the number of tweets in January 2012.

**Figure 7:**
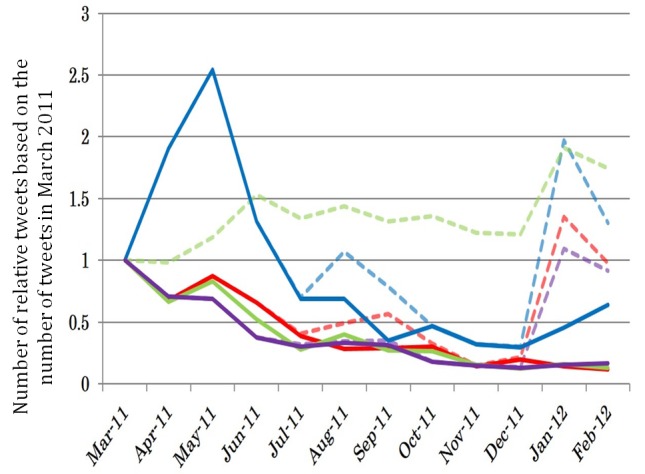
Ratio of the number of tweets in each month based on the number of tweets in each district at the time of the Fukushima Nuclear Plant disaster.

Figure 7 presents the trends in each district, excluding tweets posted automatically, based on the number of tweets at the time of the Fukushima Nuclear Plant disaster outbreak.

In districts 2 (red solid line), 3 (green solid line), and 4 (purple solid line), the number of tweets decreased to half of that in March by July (4 months after the Fukushima Nuclear Plant disaster outbreak). The same decrease was seen in district 1 (blue lines), but in September or half a year after the Fukushima Nuclear Plant disaster outbreak. The ratio of the number of tweets continued to decrease until December, although this trend did not apply to district 1 (blue solid line) in January 2012.

## Discussion

### Increase in Tweets on Radiation in Each District in May 2011

In 3 districts (except district 4), the number of tweets increased in May, or 2 months after the Fukushima Nuclear Plant disaster outbreak. Especially in district 1, the number of tweets increased to approximately 2.5 times compared with the March 2011 numbers. This trend coincided with the reports in May on the meltdown at Fukushima Nuclear Power Station No. 1 that relayed information on the high concentration of water contamination from a part of the soil in Fukushima. The main radioactive nuclide released from the nuclear power plant was iodine-131, which can increase the risk of thyroid cancer as epidemiologically demonstrated just after the Chernobyl disaster [17]. The news also covered the death of an employee on site at the Fukushima Nuclear Power Plant. These news reports may have stimulated civic interest in radiation, evidenced by the increase in number of tweets on the leak of highly concentrated radioactive material outside of Fukushima Nuclear Power Plant, on the geographic distance of the nuclear workstations, and on the health risks or potential fatality of radiation in the Fukushima Nuclear Plant disaster.

### Increase in Number of Tweets on Radiation Doses in January in Each District

Automatically posted tweets increased in each district in January, and many of them indicated radiation doses in certain areas. This trend may indicate the uneasiness of the public regarding radiation, which had been shown to be fatal to humans. A survey conducted during March 12-15, 2012 on the internet with 1793 parents with small children living in the Tohoku region, Kanto region, and Kansai region reported that a total of 73.3% of the surveyed Japanese parents experienced anxiety after the Fukushima Nuclear Plant disaster, and 52.7% of parents in the Fukushima Prefecture experienced “strong anxiety” that was higher than that reported from other regions [18]. A survey to quantify emotional responses for 284 British nationals in Japan reported that 16% met the criteria for distress, 29.7% reported high anxiety relating to the incident, and 30.4% reported high anger [19]. Therefore, the citizens sought information on radiation in their area of residence for their peace of mind.

According to the needs of the citizens in each district, municipalities began to measure the radiation dose, and the results were transmitted through various media. As a result, tweets on radiation dose increased.

### Limitations of the Study

This study has 3 limitations, as detailed below.

#### Civic Movement

The study period was set as the year after the disaster occurred. In this period, the entry and exit of people happened frequently; there was fluidity in the tweeting population. Refugees were moved to shelters; volunteers entered disaster-affected areas, and nuclear workers were brought into the plant and its environs. In other words, the places where citizens lived could be different from the places where they posted tweets, such as their workplace.

Immediately after the disaster, migration of citizens is taken into consideration as it is considered that there are not many citizens flowing in and out. However, because it is thought that citizens were flowing in and going out over more than 1 month, it is not possible to consider this point in this research; thus, we think that it is necessary to consider countermeasures.

#### Number of Twitter Users

We used the total population of 47 prefectures to calculate the number of tweets per 100,000 people in each district. However, the utilization ratio on Twitter differs according to age and is not equal. Therefore, the differences in the age composition of the population in each district generated deviation, and the ratio of the number of tweets per 100,000 people in each district may be not representative of the entire population. It would be necessary to consider the age composition in each district in a future study.

#### Number of Tweets With Geographic Information

The extracted 45,829 tweets with latitude and longitude information represent a small fraction of the 18 million tweets on radiation. Furthermore, the retweets were not given special attention. In future work, retweets on Twitter merit investigation, especially the relationship between original tweets and retweets, to show which tweets attracted public interest with respect to the need for information on radiation. It is difficult to increase the number of tweets including latitude and longitude information. We believe that we can gather more data by collecting information on latitude and longitude using information sent through other SNS and analyzing it along with Twitter data. In investigating the concern, we think that it is necessary to analyze the degree of impression of information on radiation and perform a regionality analysis on ambiguity; thus, we would like to analyze emotions as well. It is also necessary to analyze how the interest spreads. In the future, we also need to investigate the retweet information, which is the information spreading function of Twitter, and analyze the communication of medical information on Twitter.

The existence of not only accurate medical information but also erroneous medical information on the Web may hinder accurate medical information from being obtained quickly in the event of a disaster. In this study, tweet information including the phrases “radiation,” “radioactivity,” and “radioactive substance” within the target period was analyzed for the change over time of the tweet number. However, it seems that the information includes erroneous medical information. How this kind of information spreads cannot be clarified in this research. We would like to clarify how information among users will spread by analyzing retweet information in the future.

### Conclusion

The purpose of this study was to reveal how the dissemination of information on radiation changed within the year immediately after the first Fukushima Nuclear Plant disaster. District 1, or the district closest to the disaster site, showed the highest frequency of related tweets 2 months after the disaster (up to June 2011). In districts outside district 1, a high volume of radiation-related tweets was found only in March 2011, after which information sharing on this aspect decreased gradually.

The number of tweets per 100,000 people became half of that on March 2011 3 or 4 months after the Fukushima Daiichi Nuclear Plant disaster in 3 districts except district 1 (Fukushima Prefecture 9); the number became half in Fukushima Prefecture half a year later.

## Abbreviations

SNS: social network services
